# Catalytic inhibition of topoisomerase II by a novel rationally designed ATP-competitive purine analogue

**DOI:** 10.1186/1472-6769-9-1

**Published:** 2009-01-07

**Authors:** Patrick Chène, Joëlle Rudloff, Joseph Schoepfer, Pascal Furet, Peter Meier, Zhiyan Qian, Jean-Marc Schlaeppi, Rita Schmitz, Thomas Radimerski

**Affiliations:** 1 Department of Oncology, Novartis Institutes for BioMedical Research, Basel, Switzerland; 2 Global Discovery Chemistry, Novartis Institutes for BioMedical Research, Basel, Switzerland; 3 Biologics Center, Novartis Institutes for BioMedical Research, Basel, Switzerland

## Abstract

**Background:**

Topoisomerase II poisons are in clinical use as anti-cancer therapy for decades and work by stabilizing the enzyme-induced DNA breaks. In contrast, catalytic inhibitors block the enzyme before DNA scission. Although several catalytic inhibitors of topoisomerase II have been described, preclinical concepts for exploiting their anti-proliferative activity based on molecular characteristics of the tumor cell have only recently started to emerge. Topoisomerase II is an ATPase and uses the energy derived from ATP hydrolysis to orchestrate the movement of the DNA double strands along the enzyme. Thus, interfering with ATPase function with low molecular weight inhibitors that target the nucleotide binding pocket should profoundly affect cells that are committed to undergo mitosis.

**Results:**

Here we describe the discovery and characterization of a novel purine diamine analogue as a potent ATP-competitive catalytic inhibitor of topoisomerase II. Quinoline aminopurine compound 1 (QAP 1) inhibited topoisomerase II ATPase activity and decatenation reaction at sub-micromolar concentrations, targeted both topoisomerase II alpha and beta in cell free assays and, using a quantitative cell-based assay and a chromosome segregation assay, displayed catalytic enzyme inhibition in cells. In agreement with recent hypothesis, we show that BRCA1 mutant breast cancer cells have increased sensitivity to QAP 1.

**Conclusion:**

The results obtained with QAP 1 demonstrate that potent and selective catalytic inhibition of human topoisomerase II function with an ATP-competitive inhibitor is feasible. Our data suggest that further drug discovery efforts on ATP-competitive catalytic inhibitors are warranted and that such drugs could potentially be developed as anti-cancer therapy for tumors that bear the appropriate combination of molecular alterations.

## Background

Topoisomerase type II is an ATPase of the GHKL (gyrase, Hsp90, histidine kinase, MutL)-family that is evolutionary conserved in eukaryotes and essential for chromosome segregation during mitosis [1]. Mammals express two topoisomerase type II isoforms, alpha and beta, which are highly homologous but display differences in expression and in sub-cellular localization at the time of mitosis [2]. Like topoisomerases type I and III, the type II enzyme can remove topological constraints on DNA. However, it is the only enzyme that is capable of decatenating intertwined chromatids. Catenations in sister chromatids arise during DNA replication and must be removed to allow faithful chromosome segregation during anaphase [3]. Topoisomerase II is a homodimer that clamps onto two DNA double strands upon ATP binding to the amino-terminal ATPase domains. Subsequently, the enzyme transiently cleaves one of the DNA double strands and, using the energy derived from ATP hydrolysis, transports the second DNA double strand through the gap. The cleaved strand is rapidly religated and the DNA strands are released from the enzyme upon hydrolysis of the second molecule ATP and dissociation of ADP molecules [3,4]. Drugs that interfere with topoisomerase II function have been developed as antitumor agents and are in clinical use already for decades [5]. However, the vast majority of these drugs act by stabilizing the state in which the enzyme has introduced the DNA double strand break and induce a so-called cleavable complex [6,7]. Consequently, tumor cell death is triggered by the substantial DNA damage elicited by the so-called topoisomerase II poisons. Despite broad antitumor activity, the use of topoisomerase II poisons as cancer chemotherapy is limited by a narrow therapeutic window as concomitant damage to healthy cells and tissues is almost inevitable [8].

Drugs that inhibit topoisomerase II function without inducing cleavable complexes are termed catalytic inhibitors [9]. These drugs are thought to impede a step in the catalytic cycle that precedes DNA double strand scission and exert their antiproliferative effects by depleting the essential enzymatic function. Such drugs are thought to mainly affect cells that are committed to undergo mitosis and recent data suggest that tumor cells with defects in certain checkpoint control mechanisms might be particularly sensitive to catalytic inhibitors [10]. It has been proposed that cells employ two distinct checkpoint mechanisms in G_2 _and M phase, respectively, in response to catalytic topoisomerase II inhibition. In the G_2 _phase of the cell cycle a "DNA-decatenation checkpoint" is triggered that delays entry of cells into mitosis [11]. This checkpoint was shown to be caffeine sensitive and dependent on ATM/ATR (ataxia-telangiectasia mutated/ATM- and Rad3-related), BRCA1 (breast cancer gene 1) and the WRN (Werner syndrome gene) helicase [12-14]. Interestingly, in one study it was found that a fraction of lung cancer cell lines were hypersensitive toward the bisdioxopiperazine catalytic topoisomerase II inhibitor ICRF-193 [13]. The hypersensitive cells failed to activate ATM and to delay entry into mitosis upon incubation with ICRF-193. Furthermore, recent evidence suggests that cells arrest in metaphase upon depletion of topoisomerase II or treatment with the catalytic inhibitor ICRF-193 [15,16]. The metaphase arrest was shown to be distinct from the spindle assembly checkpoint but nonetheless to be dependent on the checkpoint protein Mad2 [15,17]. These data suggest that it may be possible to develop catalytic inhibitors of topoisomerase II as anticancer therapy to target tumors with the appropriate molecular alterations [10].

The bisdioxopiperazines inhibit the catalytic cycle of topoisomerase II by binding in proximity of the ATP binding site and trapping the enzyme as a closed clamp on DNA [18]. Alternatively, the catalytic cycle can be halted by drugs that dock into the ATP-binding pocket and prevent nucleotide binding. Drugs that target bacterial DNA-gyrase were shown to function by this mechanism of action and have been developed as antibiotics [19]. Notably, recent data suggest that this approach should also be applicable to the eukaryotic enzyme [20-22]. Starting from a homology model derived from DNA-gyrase and subsequently using the yeast [18] and more recently the human topoisomerase II ATPase domain crystal structures [23] we have undertaken structure-based drug discovery efforts on this exciting target with the aim of identifying ATP-competitive catalytic inhibitors. Relying on an enzymatic ATPase assay, these efforts have led to the discovery of novel designed purine diamino analogues as potent ATP-competitive catalytic inhibitors of topoisomerase II. The quinoline aminopurine 1 (QAP 1) inhibited topoisomerase II ATPase activity as well as the decatenation reaction and targeted both topoisomerase II alpha and beta isoforms in cell-free assays. Importantly, QAP 1 displayed catalytic enzyme inhibition in cells as shown by antagonism of doxorubicin-induced DNA damage and aberrant chromosome segregation. Finally, we show that breast tumor cells with a mutation in BRCA1 have increased sensitivity towards QAP 1 as compared to cells reconstituted with BRCA1.

## Results

### Screening for ATP-competitive Topoisomerase II Inhibitors using Molecular Modeling combined with an ATPase Assay

**Figure 1:**
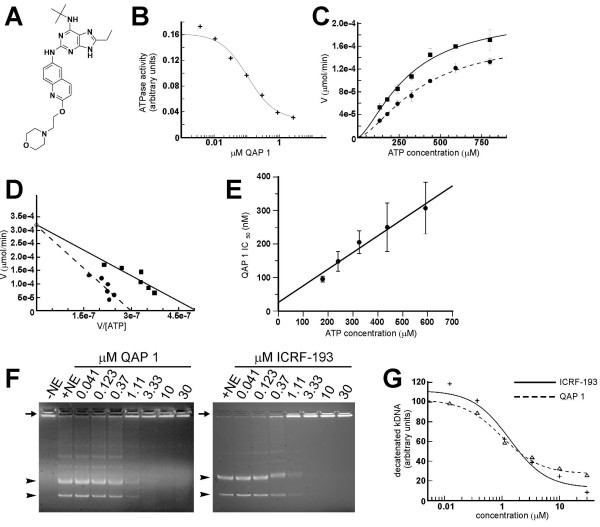
**Identification of QAP 1 as an ATP-competitive catalytic inhibitor of topoisomerase II**. (A) Chemical structure of the substituted purine analogue QAP 1. (B) Concentration-dependent inhibition of topoisomerase II ATPase activity by QAP 1. (C, D) Kinetic analysis of DNA-dependent topoisomerase II ATPase activity with vehicle or QAP 1 (130 nM) showing product formation in dependence of substrate concentration (C) and Eadie-Hofstee plot (D). (E) Plot representing the IC_50_s determined in the presence of different ATP concentrations. (F) Inhibition of topoisomerase II-mediated DNA decatenation in vitro by QAP 1 as compared to ICRF-193. Assays were carried out with topoisomerase II from nuclear extracts (NE). The arrow marks the position of catenated kDNA substrate and the arrowheads designate the positions of decatenated kDNA topoisomerase II products, nicked circular and closed circular minicircles, respectively. (G) IC_50 _determination of the inhibition of DNA decatenation by QAP 1 and ICRF-193, respectively.

Based on homology structural models of the target, a purine scaffold was designed interactively using the Macromodel modeling software [24]. The designed purine scaffold was subsequently used as a substructure query to search the Novartis compound collection. Around 50 molecules were retrieved in this manner and screened in an ATPase assay using purified full length tagged human topoisomerase II alpha. Screening this set of compounds resulted in two micromolar hits that were further optimized by structure-based design (manuscripts in preparation). Submicromolar activity with an IC_50 _of 432 ± 30 nM in the ATPase assay was obtained with the purine analogue 3 that is substituted with an ethyl group at position C6, a tert-butyl group at position N^6 ^and containing a benzothiazole group in position N^2 ^(manuscripts in preparation). Introduction of a morpholino-ethoxy group in the quinoline aminopurine (QAP 1) improved activity in the ATPase assay to an IC_50 _of 128 ± 21 nM (Figure 1A, B). Subsequently, we carried out kinetic analysis with QAP 1 in order to corroborate the mode of action. When the apparent K_m _of topoisomerase II for ATP was determined in the presence of IC_50 _concentrations of QAP 1 (130 nM) an increase in the K_m _from 310 ± 6 nM to 466 ± 84 nM was observed (Figure 1C). The data were analyzed by non-linear regression analysis using the equations corresponding to competitive, uncompetitive, and mixed inhibition (see Methods) and a statistical analysis (F-test; p ≤ 0.05) was carried out to identify the best model. These studies revealed that QAP 1 is an ATP-competitive inhibitor of topoisomerase II. A linearized representation (Eady-Hofstee) of one of these experiments is depicted in Figure 1D. To confirm the mode of action of QAP 1, inhibition studies were also carried out in the presence of different concentration of the inhibitor at different ATP concentrations and the IC_50 _values were determined. Figure 1E shows that, according to the Cheng-Prusoff equation for a linear competitive inhibitor [25], the IC_50 _of QAP 1 increases in a linear fashion with increasing ATP concentrations. These data, together with the results presented in Figure 1D suggest that QAP 1 is an ATP-competitive inhibitor. Next, QAP 1 was tested against a panel of kinases in a radiometric assay [26]. With the exception of subtle inhibition of the Ret and c-Src tyrosine kinases no effect of the compound was observed on other kinases in the panel, even at the highest concentration tested (Table 1). Thus, QAP 1 is a relatively potent inhibitor of topoisomerase II ATPase function with fairly good selectivity over various serine/threonine/tyrosine protein kinases. To test if QAP 1 inhibited topoisomerase II DNA strand passage activity, nuclear extracts of HL-60 leukemic cells were incubated with catenated kDNA in the presence of increasing concentrations of QAP 1. In this functional assay QAP 1 was found to inhibit DNA decatenation with a mean IC_50 _of 770 nM (Figure 1F, G), which was in the range of the values measured with ICRF-193 [27], a non-ATP competitive catalytic inhibitor of topoisomerase II and most potent bisdioxopiperazine described to date. Similarly, in DNA decatenation assays using the purified human topoisomerase II alpha enzyme (additional file 1) a mean IC_50 _of 660 nM was determined for QAP 1.

**Table 1:** Kinase selectivity profiling of QAP 1 in radiometric filter binding assays

**Kinase**	**IC_50 _(μM)**	**Kinase**	**IC_50 _(μM)**
HER-1	> 10	FGFR3-K560E	> 10

KDR	> 10	Axl	> 10

Flt-3	> 10	Fak	> 10

IGF-1R	> 10	c-Abl	> 10

Tek	> 10	c-Abl-T315I	> 10

c-Src	1.7 ± 0.5	PKA	> 10

c-Met	> 10	CDK1/CycB	> 10

Ret	1.35 ± 0.05	PKB	> 10

Jak2	> 10	PDK1	> 10

EphB4	> 10	BRAF-V600E	> 10

QAP 1 was profiled against a panel of serine/threonine/tyrosine protein kinases. Data typically represent means of two independent experiments ± SD.

### QAP 1 Inhibits Topoisomerase II Alpha and Beta Isoforms in a Cell Free Assay

**Figure 2:**
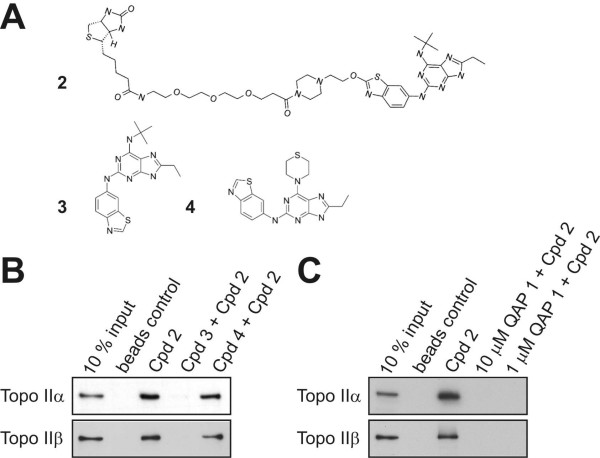
**Cell-free topoisomerase II alpha and beta interaction studies with substituted purine analogues**. (A) Chemical structures of biotinylated purine analogue 2 (compound (cpd) 2), purine analogues 3 (cpd 3) and 4 (cpd 4), respectively. (B) Binding of both topoisomerase II alpha and beta to 1 μM biotinylated purine analogue 1 and disruption of binding by parent compound 3 but not ATPase inactive compound 4 (both at 10 μM). (C) Binding of topoisomerase II alpha and beta to 1 μM biotinylated compound 1 is abolished by 10 fold molar excess and equimolar concentrations of QAP 1.

In mammals two topoisomerase II isoforms exist, topoisomerase II alpha and beta. The alpha isozyme is expressed in a cell-cycle regulated manner with highest levels of expression in G_2_/M. Topoisomerase II beta is expressed constitutively in differentiated and in proliferating cells. Although mouse knockout studies have demonstrated that topoisomerase II alpha but not beta is essential for cell proliferation [28,29], recent siRNA data suggest that the beta isoform can compensate when alpha is depleted in certain tumor cells [30]. In agreement with these findings, we have detected an antiproliferative effect and induction of polyploidy only after depletion of both topoisomerase II isoforms using RNAi in HeLa cells (data not shown). However, the proposed partial compensation by the beta isoform does not occur in all cancer cell settings [31] and could also be attributable to insufficient enzyme depletion in the alpha knockdown experiments. Nonetheless, the fact that partial compensation might arise should be taken into consideration for drug discovery efforts; that is for cancer therapy with catalytic inhibitors, inhibition of both isoforms may be required. Concurrent inhibition of topoisomerase II alpha and beta should be achievable with ATP-competitive catalytic inhibitors as the ATPase domains display a high degree of homology. To ensure that the purine scaffold targeted both topoisomerase II isoforms the biotinylated substituted purine analogue 2 was incubated with HL-60 nuclear extracts. Using the biotinylated substituted purine analogue 2 it was possible to pull down topoisomerase II alpha and beta isoforms (Figures 2A, B). The interaction was lost when the nuclear extracts were incubated with an excess of non-biotinylated parent compound, purine analogue 3 (manuscripts in preparation) but not when incubated with an excess of the substituted purine analogue 4 that was inactive in the ATPase assays (Figure 2B). Importantly, the interaction of biotinylated purine analogue 2 with both topoisomerase II alpha and beta isoforms was abolished when QAP 1 was incubated in excess or at equimolar concentrations (Figure 2C).

### QAP 1 displays Catalytic Topoisomerase II Inhibition in a Quantitative Cell-Based Assay

**Figure 3:**
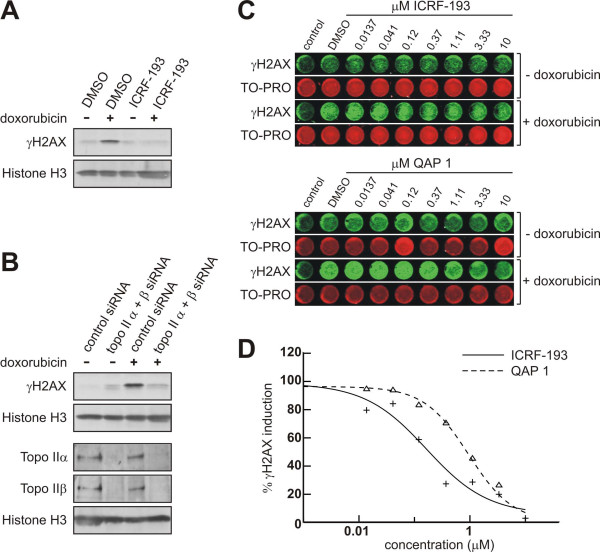
**Assessment of catalytic topoisomerase II inhibitor relative cellular potency**. (A) Catalytic inhibition of topoisomerase II by ICRF-193 prevents induction of DNA damage by doxorubicin in HL-60 cells as assessed by γH2AX Western blotting. Histone H3 levels are shown as loading control. Both drugs were incubated at 5 μM. Cells were pretreated with ICRF-193 for 30 minutes prior to addition of doxorubicin for 2 hours. (B) Induction of γH2AX by doxorubicin (5 μM, 2 hours treatment) is abolished in HeLa cells in which topoisomerase II alpha and beta have been depleted by siRNA. Histone H3 levels are shown as loading control in the Western blots. (C) Representative examples of the γH2AX in-cell western assay in 96 well format showing suppression of doxorubicin-induced γH2AX signal by increasing concentrations of ICRF-193 and QAP 1, respectively. Control designates non-specific γH2AX signal detected in wells where primary antibody was omitted to correct for background. TO-PRO was used to normalize for cell numbers. (D) IC_50 _determination of the inhibition of doxorubicin-induced γH2AX signal by QAP 1 and ICRF-193, respectively.

Although the enzymatic and biochemical cell free experiments show that QAP 1 is a catalytic inhibitor of topoisomerase II-mediated DNA decatenation and targets both enzyme isoforms it is important to assess if the compound can inhibit topoisomerase II in the cellular context. If cells are pretreated with catalytic topoisomerase II inhibitors the amount of active enzyme on chromatin is sharply reduced. It is by this mechanism that catalytic inhibitors are thought to antagonize subsequent treatment with topoisomerase II poisons [32]. DNA damage in the form of double strand breaks is recognized by cellular surveillance mechanisms and "tagged" at the sites surrounding the break by posttranslational modification of histone H2AX in the form of Ser139 phosphorylation (γH2AX) [33]. In a first step, it was confirmed that the DNA damage readout (γH2AX) induced by doxorubicin was suppressed by pretreatment of cells with the catalytic inhibitor tool compound ICRF-193 (Figure 3A). Next, we corroborated that the induction of γH2AX by doxorubicin was strictly dependent on the target protein. Since both topoisomerase II isoforms contribute to DNA damage elicited by anthracyclines [27], HeLa cells were transfected either with control siRNA or co-transfected with topoisomerase II alpha and beta siRNAs. After an incubation period of 72 hours the cells were treated with doxorubicin for two hours and then extracted for analysis of histones by Western blotting. In cells depleted of both isoforms the induction of γH2AX signal by doxorubicin was substantially blunted, demonstrating that the DNA-double strand breaks created by this drug in this experimental setting require topoisomerase II (Figure 3B). Thus, the γH2AX readout can be used to profile novel catalytic inhibitors of topoisomerase II for cellular activity and to assess relative potency. In order to extend the method for higher throughput and a quantifiable format we adapted the assay to In Cell Western in 96 well plates [34]. Cells were treated with increasing concentrations of either ICRF-193 or QAP 1 for 2.5 hours or pretreated with the same dose range of either drug for 30 minutes prior to addition of 1 μM doxorubicin for 2 hours. Cells were fixed, stained and then the γH2AX and TO-PRO signals were detected and quantified with the Odyssey reader. ICRF-193 suppressed the doxorubicin-induced γH2AX signal with a mean IC_50 _in the range of 0.2 μM (SD ± 0.11 μM, n = 3) and QAP 1 displayed an IC_50 _value in the range of 1.1 μM (SD ± 0.07 μM, n = 2) (Figure 3C, D). Neither catalytic inhibitor elicited detectable γH2AX induction by itself using this assay, as assessed by comparing γH2AX signals with the signals in DMSO treatment controls. However, we cannot rule out that detection of subtle DNA damage in the form of few DNA double strand breaks per cell is missed in the In Cell Western assay, as several groups have reported that bisdioxopiperazines can damage DNA [35,36] and high concentrations of ICRF-193 were shown to induce γH2AX foci [37]. Taken together, QAP 1 targets topoisomerase II in cells, however, the drug is less potent in the cellular context as compared to ICRF-193.

### Perturbation of Chromosome Segregation by QAP 1 in a Functional Cellular Assay

**Figure 4:**
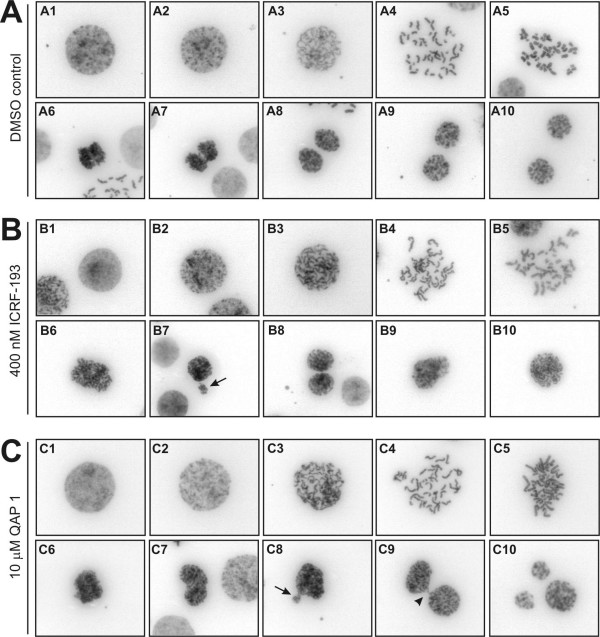
**Analysis of chromosome segregation in HL-60 cells by chromosome spreading**. HL-60 cells were synchronized to the G_1_/S boundary by aphidicolin treatment for 16 hours and then released into fresh medium. After 6 hours release, the cells were treated as indicated below for 2 hours prior to preparation of chromosome spreads. (A) Cells treated with DMSO vehicle and 1 mM caffeine. (B) Cells treated with 400 nM ICRF-193 and 1 mM caffeine. (C) Cells treated with 10 μM QAP 1 and 1 mM caffeine. The following mitotic stages are shown: Late G2 (A1, B1 and C1), early prophase (A2, B2 and C2), prophase (A3, B3 and C3), prometaphase (A4, B4 and C4), metaphase (A5, B5 and C5), anaphase (A6, A7, B6, C6 and C7), telophase (A8–10, B7–B10 and C8–C10). Note, that each image represents a different cell. Arrows depict cells with evidence of chromosome malsegregation, arrow head depicts anaphase bridging.

Early work in yeast demonstrated that topoisomerase II function is essential at the time of mitosis for chromosome segregation [38,39]. To assess if topoisomerase II inhibition by QAP 1 qualitatively resulted in aberrant chromosome segregation in cells, spreads were prepared at the time of mitosis. HL-60 cells were synchronized to the G_1_/S boundary by aphidicolin treatment and released into fresh medium. After 6 hours most cells had entered the G_2 _phase and mitosis was occurring by 8 hours as assessed by FACS analysis and measuring the mitotic index (data not shown). As a first step, ICRF-193 was used to monitor the consequences of topoisomerase II inhibition in this cellular system. When cells had been released for 6 hours and then treated with 1 μM ICRF-193 and caffeine (to override the DNA decatenation checkpoint) for 2 hours no normal anaphases or telophases could be observed (data not shown), indicating that the catalytic topoisomerase II inhibitor had prevented DNA decatenation and timely normal chromosome segregation. This is seemingly consistent with recent evidence showing that topoisomerase II depletion or ICRF-193 treatment can delay cells in metaphase up to several hours in a Mad2 dependent manner before they attempt cytokinesis [15,16]. However, at lower concentrations of ICRF-193 (400 nM) cells could be identified that had progressed beyond metaphase and anaphase. In the absence of a more detailed mechanistic understanding of the Mad2-dependent topoisomerase II metaphase checkpoint we can only speculate that this checkpoint might be leaky in HL-60 cells, allowing slippage and progression into anaphase despite compromised topoisomerase II function. Alternatively, checkpoint activation might require levels of catenated DNA in mitosis to exceed a certain threshold, since cells normally enter mitosis with residual sister chromatid catenations that are only resolved at the time of metaphase to anaphase transition [40] and during anaphase [41]. In the presence of low ICRF-193 concentrations anaphases looked abnormal whereas in control treated cells two separating groups of chromatin were readily observable (cf. Figure 4A, B panels 6). Presumably the abnormal anaphases are the consequence of anaphase bridging due to incomplete DNA decatenation. Cells were detected that successfully managed to progress to telophase as seen by the reconstitution of two adjacent nuclei containing decondensing DNA without detectable signs of chromosome-bridging (Figure 4B panel 8) but some cells also displayed obvious signs of chromosome miss-segregation (Figure 4B panel 7) and cells were observed that failed entirely to segregate their chromosomes and reconstituted tetraploid nuclei (Figure 4B panels 9, 10). Importantly, when cells were treated in G_2 _with 10 μM QAP 1 mitosis was also severely perturbed during anaphase, which is consistent with topoisomerase II inhibition (Figure 4C). Furthermore, although sister chromatid resolution occurred, metaphase chromosomes in QAP 1 treated cells consistently appeared slightly less condensed as compared to control treated cells (cf. Figure 4A, C panels 5). This latter finding is in agreement with topoisomerase II depletion studies and with earlier work using bisdioxopiperazines that imply a role for topoisomerase II in the final compaction of chromosomes [30,31]. We believe that the concentrations of ICRF-193 and QAP 1 that evidently perturbed mitotic progression at anaphase and beyond relate relatively well with intracellular target inhibition and ensuing antiproliferative effects. Indeed, in proliferation assays over 72 hours the half-maximal growth inhibitory concentration (GI50) was determined as 250 nM for ICRF-193 and 8 μM for QAP 1 in HL-60 cells.

### BRCA1 Mutant Breast Cancer Cells Display Increased Sensitivity to both Non-ATP-Competitive and ATP-Competitive Catalytic Topoisomerase II Inhibition

**Figure 5:**
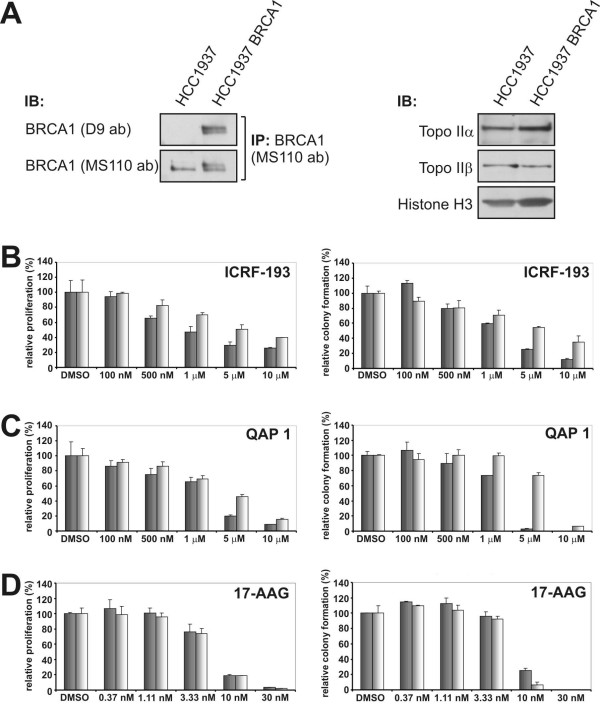
**BRCA1 mutant HCC1937 cells display increased sensitivity to catalytic topoisomerase II inhibition by ICRF-193 and QAP 1**. (A) Left panel shows the immunoprecipitation (IP) of BRCA1 from HCC1937 BRCA1 mutant and HCC1937 BRCA1 reconstituted cells using an antibody that recognizes an internal epitope. In the Western blot (IB) using an antibody directed towards BRCA1 carboxyl-terminus the protein is only detected in IPs from the reconstituted cell line. Right panel: Levels of topoisomerase II alpha and beta in nuclear extracts of HCC1937 BRCA1 mutant and HCC1937 BRCA1 reconstituted cells. Histone H3 was used as a loading control. (B-D) Assessment of relative proliferation and colony formation in representative clonogenic growth assays with HCC1937 BRCA1 mutant (grey bars) and HCC1937 BRCA1 reconstituted (white bars) cells following continuous treatment for 9 days with ICRF-193, QAP 1 and 17-AAG, respectively.

Given the findings that QAP 1 targets topoisomerase II catalytic function in cells we explored the action of the drug in a model of BRCA1 mutation. It has previously been shown that G_2 _DNA decatenation checkpoint deficiency can confer hypersensitivity to the catalytic topoisomerase II inhibitor ICRF-193 [13] and that ATR/BRCA1 are required to enforce this checkpoint [12]. BRCA1 mutant HCC1937 breast cancer cells were previously shown to be deficient in the DNA decatenation checkpoint and that this checkpoint is restored in HCC1937 cells with reconstituted wild type BRCA1 expression [12]. In addition, BRCA1 appears to be important for regulating topoisomerase II DNA decatenation activity [42]. The effects of ICRF-193 and QAP 1 on BRCA1 mutant and BRCA1 reconstituted HCC1937 cells were assessed in clonogenic growth assays. Immunoprecipitation and Western blotting with a combination of different BRCA1 antibodies was used to confirm that the HCC1937 cells expressed mutant carboxyl-terminally truncated BRCA1 and that the BRCA1 reconstituted HCC1937 expressed the full-length protein (Figure 5A). Analysis of nuclear extracts revealed that both cell lines expressed comparable levels of topoisomerase II alpha and beta (Figure 5A). In clonogenic growth assays, we observed that BRCA1 mutated cells were somewhat more sensitive towards ICRF-193 as determined by measuring relative proliferation using the WST-1 reagent, a tetrazolium salt that is cleaved in mitochondria of metabolically active cells, and by counting colony numbers after crystal violet staining (Figure 5B). Encouragingly, QAP 1 also displayed a differential antiproliferative effect on BRCA1 mutant versus BRCA1 reconstituted cells and this effect was most evident at a concentration of 5 μM (Figure 5C). At this dose of the compound a striking suppression of colony formation of BRCA1 mutant cells was seen. In contrast, the Hsp90 inhibitor 17-AAG affected growth of BRCA1 mutant and BRCA1 reconstituted cells to a similar extent (Figure 5D), suggesting that the effects seen with the catalytic topoisomerase II inhibitors were not due to a general hypersensitivity of BRCA1 mutant cells towards anti-proliferative agents.

## Discussion

The novel rationally designed purine analogue QAP 1 was found to inhibit human topoisomerase II DNA-dependent ATPase activity and DNA decatenation in vitro with submicromolar IC_50_s and to inhibit the enzyme with an ATP-competitive mode of action. Although purine analogues have recently been described as catalytic inhibitors of topoisomerase II [21,22], to our best knowledge QAP 1 represents the most potent and selective example of this chemotype described to date. Interestingly, topoisomerase II dimers consisting of one wild-type and one ATPase dead molecule have been found to still be capable of carrying out DNA strand passage reactions in vitro [43]. This suggests that ATP-competitive inhibitors may need to attain intracellular concentrations that saturate both ATP-binding sites of the topoisomerase II dimer for efficient inhibition of DNA decatenation, whereas a single bisdioxopiperazine molecule binds and bridges the ATP-associated dimer molecules [18].

In vitro QAP 1 displayed greater than 10 fold selectivity over a standard panel of kinases, although some inhibition of the Ret and Src tyrosine kinases was observed. Further optimization will be required to reach low nanomolar potency toward topoisomerase II while improving selectivity over kinases. The recently described crystal structure of the human topoisomerase II ATPase domain is expected to aid such drug discovery efforts [23]; co-crystallization with purine scaffolds will be crucial to corroborate binding mode and provide further impetus for lead optimization.

Cellular activity of QAP 1 was demonstrated by antagonism of topoisomerase II poison-induced DNA damage and aberrant chromosome segregation, even though ICRF-193 was more potent in both of these assays. On the other hand, in the clonogenic growth assays QAP 1 and ICRF-193 affected BRCA1 mutant cells to a comparable extent at a concentration of 5 μM. Although the bisdioxopiperazine class catalytic inhibitors of topoisomerase II are used for the treatment of adult T-cell leukemia in Japan, their use in other cancer indications has been limited to the modulation of anthracycline-related cardiotoxicity [9]. A key issue in the development of catalytic inhibitors of topoisomerase II is that, although the enzyme is clearly druggable, it is currently not obvious which tumors bearing which kind of molecular alterations will respond particularly well to such drugs and what the effects on normal cells and stem cells will be [44]. It is anticipated that catalytic inhibitors will show less side effects compared to topoisomerase II poisons but will also display a narrower anti-tumor spectrum. Importantly, concepts for exploiting the anti-proliferative activity of catalytic topoisomerase II inhibitors based on molecular characteristics of the tumor cell have recently started to emerge [13]. The discovery of checkpoint mechanisms in G_2 _and M phase that appear to protect cells transiently from progressing into and through mitosis in the absence of catalytic topoisomerase II function is a promising starting point [12,15,16]. The finding that BRCA1 mutant cells displayed slightly increased sensitivity to QAP 1 supports a role of the protein in the response to catalytic topoisomerase II inhibition. Interestingly, BRCA1 appears to have a dual link to topoisomerase II in that it is required (i) to enforce the DNA decatenation checkpoint and (ii) to directly regulate topoisomerase II activity by ubiquitination [12,42]. Takahashi and colleagues have observed remarkable hypersensitivity of a subset of lung cancer cells to ICRF-193. The hypersensitive cell lines lacked DNA decatenation checkpoint function and appeared to have impaired ATM activation in response to ICRF-193 treatment [13]. However, these cannot be the only criteria since it was found that overriding the DNA decatenation checkpoint with caffeine was not sufficient to render non-sensitive cells hypersensitive to the catalytic inhibitor [13]. Thus, more research will be required to unravel the molecular alterations predictive of a particularly good response to catalytic topoisomerase II inhibitors and the ICRF-193 hypersensitive cell lines alluded to above could be invaluable tools for this purpose. Finally, given the findings of several groups that bisdioxopiperazines can interfere with DNA metabolism and damage DNA [35-37] and that stem and progenitor cells have an inefficient decatenation checkpoint [44], it will be important to determine the effects of ATP-competitive catalytic topoisomerase II inhibitors in these settings. The potential therapeutic value of potent and selective ATP-competitive inhibitors will also be determined by their impact on non-tumor cells versus sensitive tumor cells in pre-clinical models.

## Conclusion

In the present study, we describe a novel purine diamine analogue, termed QAP 1, which emerged from molecular modeling efforts as a potent catalytic inhibitor of topoisomerase II alpha and beta. The inhibitor blocks topoisomerase II ATPase and decatenation activity in biochemical assays and depletes enzyme function in cellular systems. In particular, we show that QAP 1 antagonizes anthracycline-mediated DNA damage as assessed by suppression of γH2AX induction and perturbs chromosome segregation. The inhibitor QAP 1 represents a promising starting point for lead optimization campaigns and further drug discovery efforts. Importantly, the ATP-competitive catalytic inhibitor also represents a novel tool, with distinct mechanism of action as compared to bisdioxopiperazines, to assess the effects of topoisomerase II inhibition in cancer cells and in normal cells. It is the prevailing view that tumor cell sensitivity to catalytic topoisomerase II inhibitors may be attributed to cell lineage and/or specific checkpoint defects. In agreement with previous analyses, the present study shows that BRCA1 mutant cells display increased sensitivity to catalytic topoisomerase II inhibitors. However, many questions remain regarding the checkpoint mechanisms postulated to monitor DNA topology in G_2 _and M cell cycle phases, respectively. ATP-competitive catalytic topoisomerase II inhibitors such as QAP 1 could be instrumental to screen tumor cell lines and, combined with genetic as well as molecular studies, help identify tumor settings with increased susceptibility.

## Methods

### Chemical synthesis and reagents

Quinoline aminopurine 1 (QAP 1), biotinylated purine analogue 2, purine analogues 3 and 4 (detailed synthesis will be described elsewhere), 17-AAG and ICRF-193 (BIOMOL Research Laboratories Inc., Plymouth Meeting, PA) were dissolved in DMSO to yield 10 mM final concentration and stored at -20°C. Doxorubicin (BIOMOL Research Laboratories Inc., Plymouth Meeting, PA) was dissolved in distilled water to yield 10 mM final concentration and stored at -20°C. All other chemical reagents were analytical grade quality. All cell culture reagents were purchased from AMIMED (Allschwil, Switzerland).

### Full length tagged human topoisomerase II alpha protein production

Human topoisomerase II α cDNA was cloned in three successive steps. First the 5' region of the topoisomerase II α gene upstream of the EcoR1 site was amplified by PCR using an ATCC clone (#59748) as template and primers adding a Bgl2 site at its 5' end. The purified PCR product was digested with Bgl2 and EcoR1 and inserted into pFastBac1 (Invitrogen, Carlsbad, USA) previously digested with BamH1 and EcoR1. Then, an EcoR1/Pst1 fragment of the topoisomerase II α ATCC clone was inserted into the EcoR1/Pst1 sites of the plasmid previously obtained. Finally, the 3' end of topoisomerase II α downstream of Pst1 was amplified by a two-step PCR using the ATCC clone as template and primers adding a PreScission-His6-Strep tag and a Sph1 site at the 3' end. This product was subsequently digested with Pst1 and Sph1 and inserted into the corresponding sites of the plasmid previously obtained. The sequence of the inserted gene and flanking regions was verified by DNA sequencing. Topoisomerase II α protein was expressed in Sf21 cells (Invitrogen, Carlsbad, USA) using the Baculovirus system (multiplicity of infection 2.0, 72 h infection). The cell pellet was resuspended in 15 volumes ice cold lysis buffer (50 mM Na_2_HPO_4 _pH 7.7, 500 mM NaCl, 10 mM β-mercaptoethanol, 5 mM imidazol, 100 μg/ml PMSF, 2 mM MgCl_2 _and EDTA-free protease inhibitor cocktail (Roche Diagnostics GmbH)) and homogenized using a Heidolph Diax 600 homogenizer. Following centrifugation for 60 minutes at 48000 g and filtration through a 3 μm Millipore filter the lysate was loaded on a 5 ml HisTrap HP affinity column (GE Healthcare, Sweden), pre-equilibrated in buffer A (50 mM Tris-HCl pH 8.0, 500 mM NaCl, 10 mM β-mercaptoethanol and 5 mM imidazol). The column was washed with buffer A containing 20 mM imidazole and the protein was eluted with a gradient of buffer B (250 mM imidazole in buffer A). Pooled fractions containing topoisomerase II protein were loaded on a SPX200 size exclusion column (16/20) equilibrated with 50 mM Tris HCl pH 7.7, 150 mM NaCl and 2 mM DTT, followed by loading on a 10 ml StrepTactin column (IBA, Germany) equilibrated with 100 mM Tris HCl pH 8.0, 150 mM NaCl, 2 mM β-mercaptoethanol, 1 mM EDTA. After a brief washing step, the enzyme was eluted with and stored in 100 mM Tris-HCl pH 8.0, 150 mM NaCl, 2 mM β-mercaptoethanol, 1 mM EDTA and 2.5 mM desthiobiotin. Single-use aliquots of enzyme were flash-frozen in liquid nitrogen and stored at -80°C until use.

### ATPase assay and kinetic analysis

DNA-dependent ATP hydrolysis of human topoisomerase II α was monitored by measuring the production of inorganic phosphate using acidic molybdate and malachite green [45]. Specific ATPase activity of every batch was tested in a time-course to determine optimal incubation times and enzyme concentrations within the linear range of the assay. Reactions thus contained 10 – 40 nM topoisomerase II (dimer concentration) in reaction buffer (10 mM Tris HCl pH 7.5, 175 mM KCl, 0.1 mM EDTA, 5 mM MgCl_2_, 2 mM DTT, 2.5% glycerol and 1.7 nM supercoiled pUC18). The specific activity of the human topoisomerase II α enzyme measured in the ATPase assay was 0.24 μmol PO_4_·min^-1^·mg^-1^. Compounds were initially screened at 10 μM concentration and IC_50 _values were determined if inhibition was greater than 70%. Compounds at the indicated concentrations were added as serial dilutions prepared in DMSO and preincubated for 10 minutes at 37°C on an orbital shaker. Reactions were started by the addition of 400 μM ATP (Sigma, catalog number A2383) and allowed to proceed for 30 minutes. Reactions were terminated by the addition of 200 μl malachite green reagent and the OD was read immediately at 630 nm on a SpectraMax Plus microtiter plate reader using SoftMax Pro software (Molecular Devices). For kinetic analysis, reactions were carried out in duplicate as described above either in the presence of vehicle (DMSO) or QAP 1. Following a preincubation for 5 minutes, ATP was added at the indicated concentrations to start the reactions, which were allowed to proceed for 17 minutes before stopping and reading as above. From these values the OD values obtained at each respective ATP concentration without enzyme were subtracted for background correction. The low signal to noise ratio of the malachite green assay did not permit the study of a broad range of inhibitor concentrations in competition experiments. Low inhibitor concentrations did not result in enough inhibition to clearly demonstrate the effect of the inhibitor and higher inhibitor concentrations gave very weak signals. A compromise was found by carrying out the competition experiments with a single concentration of the inhibitor that gave a good inhibition of enzyme activity while keeping a measurable signal. The equations used to fit these experiments are:

Competitive inhibition:

$$v=\frac{V_{\max}.[S]}{K_{m}.(1+\frac{\left[I\right]}{K_{iS}})+[S]}$$

Uncompetitive inhibition:

$$v=\frac{V_{\max}.[S]}{K_{m}+[S].(1+\frac{\left[I\right]}{K_{iI}})}$$

Mixed inhibition:

$$v=\frac{V_{\max}.[S]}{K_{m}.(1+\frac{\left[I\right]}{K_{iS}})+[S].(1+\frac{\left[I\right]}{K_{iI}})}$$

Each competition experiment was fitted (Grafit version 5.04 – Erithacus Software) with these 3 equations and the models that gave unrealistic inhibition constants (above 10 mM) and/or large standard errors (similar to the value of the fitted parameter) were rejected. Then, the F-test was carried out to identify the best fitting model. An alternative method was used to determine the mode of action of the inhibitor. Dose-response experiments were carried out at different ATP concentrations and the corresponding IC_50_s determined. According to the Cheng-Prusoff equation for linear competitive inhibition [25]:

$$IC_{50}=K_{i}(1+\frac{\left[ATP\right]}{K_{m}})$$

The IC_50 _of a competitive inhibitive inhibitor should increase in a linear fashion with the concentration of ATP. The measured IC_50_s were then plotted in function of the ATP concentrations and linear regression analyses were carried out. For calculation of Michaelis-Menten kinetic parameters [46] data were plotted in XLfit 4 (XLfit 4 curve fitting software for Microsoft Excel, ID Business Solutions Ltd, Guildford, Surrey, United Kingdom) and linearized according to Eadie-Hofstee. In the latter plot data points obtained at the lowest ATP concentration were omitted as these are the most error prone. Mean K_m _and V_max _values were calculated from two independent experiments.

### DNA decatenation assay

Reagents for the assay were purchased from TopoGEN (catalog number 1001–1, TopoGEN, Inc., Port Orange, FL). Topoisomerase II was extracted according to the manufacturer's instructions from nuclei of HL-60 cells (ATCC; American Type Culture Collection, Manassas, Virginia, USA) and contained both isoforms as assessed by Western blotting. Calculation of specific topoisomerase II decatenation activity was based on complete decatenation of a given amount of catenated input DNA (100 ng kDNA) by a defined amount of nuclear extract containing the alpha and beta isoforms or of a defined amount of purified enzyme in a particular amount of time. The topoisomerase II activity from nuclei decatenated 0.07 ng catenated kDNA · min^-1 ^· ng^-1 ^extract. Purified topoisomerase II alpha (see above) decatenated 0.13 ng catenated kDNA · min^-1 ^· ng^-1^. Vehicle (DMSO) or drug substance at concentrations to yield the desired end concentrations were added and samples were preincubated for 5 minutes at 37°C and 400 rpm in an Eppendorf thermomixer. Reactions were started by adding ATP (to 450 μM final concentration) to each sample and incubation was continued for 20 minutes. Reactions were terminated by placing the samples on ice and adding 4 μl stop/gel loading buffer. 20 μl of each sample were separated by 1% agarose gel electrophoresis. Gels were analyzed under a UV transilluminator and decatenated kDNA products were quantified using AlphaEaseFC (FluorChem 8900) image analysis software version 3.2.3 (Alpha Innotech, San Leandro, CA).

### Affinity precipitation

Topoisomerase II α and β were affinity precipitated with biotinylated inhibitor as follows. Exponentially growing HL-60 cells were collected on ice and incubated with hypotonic buffer (10 mM HEPES pH 7.5, 1.5 mM MgCl_2_, 10 mM KCl, 0.5 mM DTT, 0.5 mM PMSF, 1× protease inhibitor cocktail (Complete Mini, Roche Diagnostics GmbH)) for 15 minutes, then 0.6% v/v NP-40 was added and cells were disrupted using a Dounce homogenizer. Lysates were transferred to 1.5 ml Protein LoBind Eppendorf tubes and nuclei were collected after centrifugation at 8000 rpm for 10 minutes and washed twice with hypotonic buffer. To extract and release chromatin associated topoisomerase II nuclei were incubated for 30 minutes in DNAse buffer (20 mM Tris HCl pH 7.5, 5 mM MgCl_2_, 1 mM CaCl_2_, 25 mM NaCl, 0.5 mM EDTA, 1% NP-40, 1 mM DTT, 1 × protease inhibitor cocktail, 0.1 mM PMSF) containing 40 U DNAse I (Roche Diagnostics GmbH) followed by addition of twice concentrated nuclear extraction buffer (20 mM HEPES pH 7.5, 3 mM MgCl_2_, 20 mM KCl, 50 mM β-glycerophosphate, 840 mM NaCl, 1 mM EDTA, 50 mM PNPP, 1 mM DTT, 1 mM PMSF) and rotation for 1 h in the coldroom. The soluble fraction was obtained following centrifugation at 13000 rpm for 15 minutes. 25 μg nuclear extract were used for the affinity precipitation. The salt concentration was adjusted to 150 mM with nuclear extraction buffer (without NaCl) and the sample volume was toped off to 200 μl with precipitation buffer (50 mM HEPES pH 7.4, 150 mM NaCl, 25 mM β-glycerophosphate, 25 mM NaF, 5 mM EGTA, 1 mM EDTA, 15 mM PPI, 1 mM PMSF). Compounds were preincubated for 30 minutes prior addition of biotinylated purine analogue 2 for 2 h. Streptavidin agarose beads (Novagen, 69203) were washed twice with PBS, blocked for 30 minutes with PBS containing 1% BSA, washed twice with PBS and finally with precipitation buffer. 40 μl equilibrated streptavidin agarose beads were added to the sample and incubation was continued for 1 h. Beads were pelleted by centrifugation at 7000 rpm for 5 minutes and washed thrice with precipitation buffer. During the last wash the beads were transferred to a new tube and the bound fraction was released by boiling for 5 minutes in Laemmli sample buffer. Topoisomerase II α and β were detected by Western blot as described below.

### Topoisomerase II α and β siRNA

0.3 × 10^6 ^HeLa T-Rex cells (Invitrogen, Paisley, United Kingdom UK) per 6-cm plate were seeded in 2.6 ml of medium without antibiotics. The day after seeding, cells were transfected for 72 hours with stealth RNA oligos (Invitrogen, Paisley, United Kingdom UK): stealth control oligo 1: 5'ACUUCCGAUUCGUGUAACCGAC UUU3'; and stealth control oligo 2: 5'GGGCUA GUUAUAGUCGACACUGGUA3') or with stealth RNA oligos directed against topoisomerase II α and β transcripts (α1: 5'ACUCAGCCUCUUAUGUGCCAAGUUU3'; α2: GGACAACAUUUG AUCCAAGAUCUUA3' and β1: 5'GGGUGAUCUUGAUACUGCAGCAGUA3'). Lipofectamine™ 2000 was used to transfect oligos according to the manufacturer's instructions. Lipofectamine containing medium was replaced 6 hours after transfection by 3 ml of fresh culture medium. Cells were extracted in 200 μl lysis buffer (50 mM Tris-HCl, pH 7.5; 5 mM EDTA; 420 mM NaCl; 0.5% Nonidet P-40 (NP-40); 1 mM Benzamidine; 20 mM NaF and 1.5 mM MgCl_2_) freshly supplemented with 1 mM PMSF (from 100 mM stock solution in ethanol), 1 mM DTT (from 1 M stock solution) and 1× protease inhibitor cocktail. Lysates were centrifuged at maximum speed in an Eppendorf centrifuge for 10 minutes at 4°C. The nuclear pellet was resuspended in 22 μl of Laemmli sample buffer and boiled at 95°C for 5 minutes. 15 μl of nuclear extracts were subjected to 6% denaturing SDS-PAGE, transferred to PVDF membranes and probed with the anti-topoisomerase II β antibody (sc-13059, Santa Cruz Biotechnology, Santa Cruz, CA; diluted 1:1000 in PBS 0.1% Tween) using appropriate secondary antibody and enhanced chemiluminescence (ECL, Amersham Pharmacia Biotech Inc., Buckinghamshire, United Kingdom). After stripping the membrane it was re-probed with a goat anti-topoisomerase II α antibody (sc-5348, Santa Cruz Biotechnology, Santa Cruz, CA; diluted 1:2000 in PBS 0.1% Tween). Immunoreactive bands were revealed by HRP-conjugated protein G (Zymed, CA; diluted 1:3000 in PBS 0.1% Tween) using ECL reagents.

### Gamma-H2AX assay

Histone extracts of HL-60 or HeLa T-Rex cells for Western blot analysis were prepared as follows: Following treatments, medium was removed and cells were collected/washed with PBS. Cells were centrifuged for 5 minutes at 1500 rpm and 4°C and the cell pellet was resuspended in 300 μl of ice-cold hypotonic buffer (see above). Cells were transferred in a 1.5 ml Eppendorf tube and incubated for 15 minutes on ice. Thereafter, NP-40 was added to the cells to a final concentration of 0.6% (v/v) and cells were disrupted by pumping 10–15 times through a 1 ml syringe connected to a 23-gauge needle. The extract was centrifuged for 10 minutes at 8000 rpm and 4°C. The supernatant (cytoplasmic fraction) was removed and the nuclear pellet was washed once with 500 μl of ice-cold hypotonic buffer for 4 minutes at 8000 rpm. Nuclear proteins were extracted by addition of 22 μl sample buffer and boiling for 3 minutes. Samples were resolved by 15% SDS-PAGE following transfer to PVDF membrane. After blocking, membranes were incubated either with anti-γH2AX (clone JBW301, 05–636, Upstate, Charlottesville, VA, diluted 1:1000 in PBS containing 0.1% Tween 20 and 5% milk) or histone H3 (catalog number 9715, Cell Signaling Technologies Inc., Beverly, MA, diluted 1:1000 in TBS containing 0.1% Tween 20 and 5% milk) antibodies overnight at 4°C. The corresponding horseradish peroxidase conjugated secondary antibodies were diluted 1:3000 and incubated for 1 hour at room temperature and immunoreactive bands were revealed as described above.

In-Cell Western analysis was carried out as follows. HeLa T-Rex cells were seeded at the cell density of 15 × 10^3 ^cells per well in black 96-well plates (Greiner Bio-one Vacuette, St Gallen, Switzerland). The day post-seeding, cells were pretreated with increasing concentrations of either ICRF-193, QAP 1 or vehicle (DMSO) for 30 minutes followed by a 2 hours treatment with 1 μM of doxorubicin, or were only treated with the same concentrations of ICRF-193, QAP 1 or vehicle (DMSO) for 2 hours. Treatments were done in triplicate. Cells were directly fixed by adding 100 μl of 3.7% formaldehyde (diluted 1:10 in PBS from a 37% formaldehyde solution; Sigma, Buchs, Switzerland) per well for 10 minutes at room temperature. Cell membranes were permeabilized by 4 washes with 100 μl per well of PBS containing 0.25% Tween-20 for 4 minutes. Cells were blocked with 50 μl per well of Odyssey Blocking Buffer (OBB; LI-COR Biosciences GmbH, Bad Homburg, Germany) for 2 hours at room temperature with gentle agitation. 25 μl per well of γH2AX antibody (clone JBW301, catalogue number 05–636, Upstate, NY) diluted 1:500 in OBB were incubated overnight at room temperature with gentle agitation. Cells were washed 5 times with 50 μl per well of PBS containing 0.1% Tween-20 for 3 minutes and stained with 25 μl per well of goat anti-mouse InfraRedDye 800 antibody (Rocklands Immunochemicals, Inc., Gilbertsville, PA) diluted 1:800 in OBB containing 0.2% Tween-20 for 1 hour at room temperature in the dark. An additional wash with 50 μl per well of PBS containing 0.1% Tween-20 was performed before DNA staining with 50 μl per well of TO-PRO-3 iodide (642/661) (Invitrogen AG, Basel, Switzerland) diluted 1:5000 in OBB for 1 hour at room temperature in the dark. Finally, cells were washed 4 times with 50 μl per well of PBS containing 0.1% Tween-20 for 3 minutes, liquid was removed and plates were kept at 4°C until reading. γH2AX signals were read using the LI-COR Odyssey Infrared Imager with the Odyssey v1.1 software using the 800 nm channel. Blank values correspond to non-specific γH2AX signal detected in wells without primary antibody staining. The means of these values were subtracted from the γH2AX signals to correct for non-specific background. The resulting values for each sample were normalized to the DNA quantity by measuring the corresponding TO-PRO-3 iodide signals. The TO-PRO-3 iodine signals were detected by using the 700 nm channel. The percentage of γH2AX signal was determined for each concentration of ICRF193 and QAP 1 with or without doxorubicin treatment by setting the value for the treatment with vehicle and 1 μM doxorubicin to 100%. To display relative suppression of γH2AX induction and catalytic topoisomerase II inhibition in cells the values measured in the presence of catalytic inhibitor were subtracted from the corresponding value measured at the same concentration of catalytic inhibitor but in the presence of 1 μM doxorubicin. These data were plotted in XLfit 4 (XLfit 4 curve fitting software for Microsoft Excel, ID Business Solutions Ltd, Guildford, Surrey, United Kingdom) to determine the IC_50 _values of the catalytic topoisomerase II inhibitors by using four parameter logistic calculation.

### Chromosome segregation assay

HL-60 cells were treated overnight for 16 hours with 2 μg/ml aphidicolin (Sigma-Aldrich corporation, St. Louis, MO) and released by washing twice PBS and resuspending into fresh medium. After 6 hours release the cells were treated with 1 mM caffeine (Lancaster Synthesis, Heysham, Lancashire, UK) and with catalytic topoisomerase II inhibitor or vehicle for 2 hours. Cells were washed once with PBS and incubated with 56 mM KCl hypotonic solution for 30 minutes. Cells were pelleted and carefully resuspended in Carnoy's fixative. Cells were pelleted as above, supernatant was removed and cells were resuspended again in 2 ml fixative. Chromosome spreads were prepared by dropping 50 μl cell suspension onto microscopy slides. The slides were allowed to dry and then stained for 10 seconds in Hoechst 33342 dye (Molecular Probes, Eugene, Oregon, prepared 1:1000 in PBS from 10 mg/ml stock). After drying, mounting media (Vectashield^®^, Vector Laboratories, Burlingame, CA) and a cover slip were added. Samples were observed using an immersion oil lens (100 ×, Nikon, Plan Apo VC) by fluorescence microscopy (Nikon Eclipse, E600) in the DAPI channel. Images were acquired using analySIS^® ^software (Soft Imaging System GmbH, Münster, Germany). Representative images were converted to grayscale, inverted and contrast adjusted with Adobe Photoshop. Mitotic cells were categorized as described [47].

### Clonogenic growth assay with HCC1937 cells

HCC1937 BRCA1 mutant and HCC1937 BRCA1 reconstituted breast ductal carcinoma cells were cultured in RPMI-1640 medium supplemented with 10% fetal calf serum, 2 mM L-glutamine, 1 mM sodium pyruvate, 4.5 g/l glucose and 1% (v/v) penicillin/streptomycin. Status of BRCA1 was corroborated by immunoprecipitation using 50 μg nuclear extracts and 1 μg mouse monoclonal antibody MS110 (ab16780, Abcam Inc., Cambridge, MA), which is directed towards the amino-terminus of the protein. Blots were probed with BRCA1 carboxy-terminal specific mouse monoclonal antibody D9 (sc-6954, Santa Cruz Biotechnologies, Santa Cruz, CA) and, following stripping, re-probed with antibody MS110. For assays, 5000 cells in 3 ml medium were seeded per well of 6 well plates in duplicates. The day after, vehicle (DMSO) or compounds were added. After one week medium was exchanged and vehicle or compounds were re-added for an additional two days. To assess proliferation the medium in each well was replaced with 1 ml medium containing 100 μl WST-1 reagent and incubation for 20 minutes at 37°C. Of each sample 110 μl supernatant were transferred into a 96 well plate and the OD was read at 450 nm (filter at 630 nm) on a SpectraMax Plus microtiter plate reader using SoftMax Pro software (Molecular Devices). For background correction, the blank value consisted of 100 μl medium containing 10 μl WST-1. Thereafter, colonies were stained by removing medium, washing cells once with PBS and incubation with 1 ml crystal violet solution for 30 minutes at room temperature. Cells were washed twice with PBS and colonies were counted using an Artec Counter (Model 880, Artec, Dyntatech Laboratories Inc.) with diameter cut-off set to 0.2 mm. Data were expressed as growth or colony numbers relative to vehicle control treatments (100%).

## Abbreviations

ATM: ataxia-telangiectasia mutated; ATR: ATM- and Rad3-related; BRCA1: Breast cancer gene 1; DMSO: Dimethyl sulfoxide; GHKL: gyrase, Hsp90, histidine kinase, MutL; IC_50 _: Half-maximal inhibitory concentration; PBS: Phosphate buffered saline; SD: Standard deviation; WRN: Werner syndrome gene.

## Authors' contributions

PC conceived the project, established the ATPase assay, participated in the experimental design and provided assistance with the writing of the manuscript. JR performed ATPase and decatenation assays, developed the γH2AX assay and analyzed compounds in the clonogenic growth assay. JS lead the medicinal chemistry efforts for the project. PF was responsible for the structure-based drug discovery. PM, together with JS, coordinated the medicinal chemistry activities leading to the synthesis of QAP 1. ZQ performed the affinity precipitation experiments. RS prepared the topoisomerase II alpha expression construct. JMS was responsible for the expression and purification of topoisomerase II protein. TR was responsible for experimental design of the study, supervised JR and ZQ, carried out kinetic analysis as well as the chromosome segregation experiments and drafted the manuscript. All authors read and approved the final manuscript.

## Supplementary Material

Additional File 1 **QAP 1 inhibits DNA decatenation mediated by purified human topoisomerase II alpha**. Inhibition of purified human topoisomerase II alpha protein-mediated DNA decatenation in vitro by QAP 1. Lanes 1 and 10 (-Topo IIα): Samples in which the enzyme was omitted in the reaction. Lane 2 (+Topo IIα): DNA decatenation by purified enzyme and dose-dependent inhibition of the reaction by increasing concentrations of QAP 1 (lanes 3 – 9). Lane 11: DNA decatenation by purified topoisomerase II alpha enzyme is strictly dependent on the presence of ATP in the assay. The arrow marks the position of catenated kDNA substrate and the arrowheads designate the positions of decatenated kDNA topoisomerase II products, nicked circular and closed circular minicircles, respectively. Click here for file
