# Supplementary figures and images for: Catalytic inhibition of topoisomerase II by a novel rationally designed ATP-competitive purine analogue
Source: BMC Chem Biol. 2009 Jan 7;9:1. doi: 10.1186/1472-6769-9-1 (PMC2628638; doi:10.1186/1472-6769-9-1)

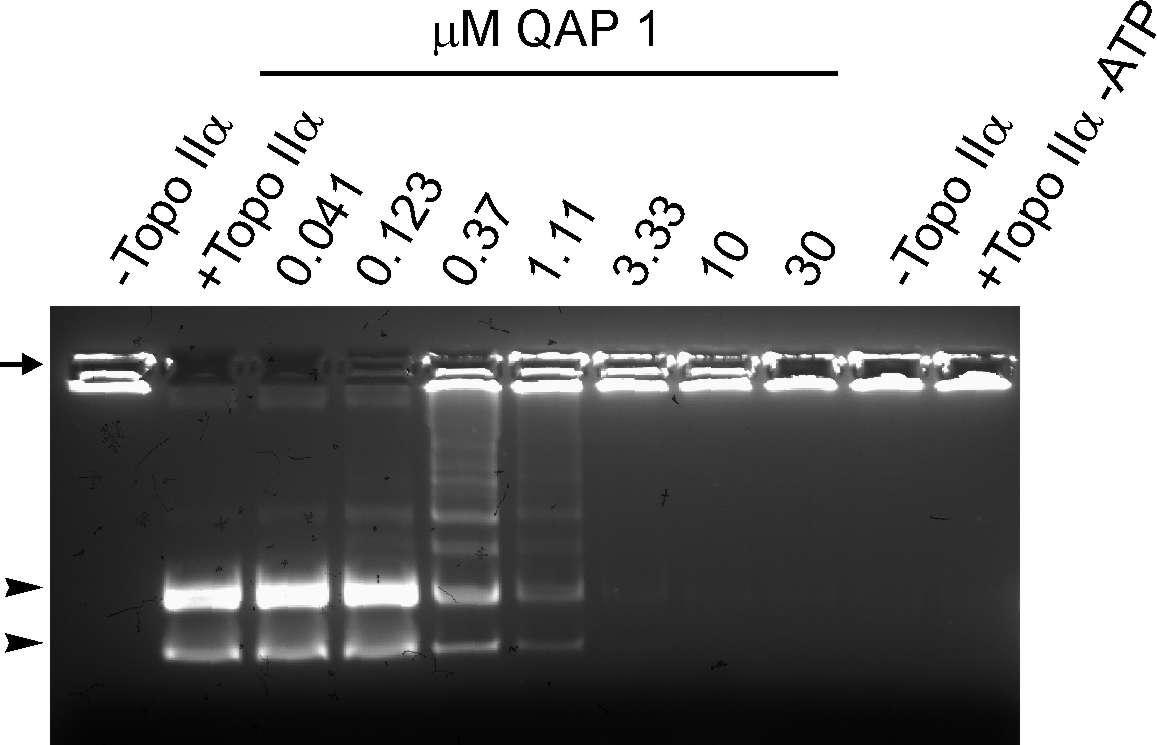

Supplement: Additional File 1 — QAP 1 inhibits DNA decatenation mediated by purified human topoisomerase II alpha. Inhibition of purified human topoisomerase II alpha protein-mediated DNA decatenation in vitro by QAP 1. Lanes 1 and 10 (-Topo IIα): Samples in which the enzyme was omitted in the reaction. Lane 2 (+Topo IIα): DNA decatenation by purified enzyme and dose-dependent inhibition of the reaction by increasing concentrations of QAP 1 (lanes 3 – 9). Lane 11: DNA decatenation by purified topoisomerase II alpha enzyme is strictly dependent on the presence of ATP in the assay. The arrow marks the position of catenated kDNA substrate and the arrowheads designate the positions of decatenated kDNA topoisomerase II products, nicked circular and closed circular minicircles, respectively. [file 1472-6769-9-1-S1.jpeg]
